# Did Medicaid expansion close African American-white health care disparities nationwide? A scoping review

**DOI:** 10.1186/s12889-022-14033-8

**Published:** 2022-08-30

**Authors:** Lonnie R. Snowden, Genevieve Graaf, Latocia Keyes, Katherine Kitchens, Amanda Ryan, Neal Wallace

**Affiliations:** 1 School of Public Health, University of California, Berkeley, USA; 2 School of Social Work, University of Texas, Arlington, USA; 3 Department of Social Work, Tarleton State University, Stephenville, USA; 4 OHSU-PSU School of Public Health, Portland State University, Portland, USA

**Keywords:** Affordable care act, Medicaid expansion, Racial disparities, Health disparities, Health policy

## Abstract

**Objectives:**

To investigate the impact of the Affordable Care Act’s (ACA) Medicaid expansion on African American-white disparities in health coverage, access to healthcare, receipt of treatment, and health outcomes.

**Design:**

A search of research reports, following the PRISMA-ScR guidelines, identified twenty-six national studies investigating changes in health care disparities between African American and white non-disabled, non-elderly adults before and after ACA Medicaid expansion, comparing states that did and did not expand Medicaid. Analysis examined research design and findings.

**Results:**

Whether Medicaid eligibility expansion reduced African American-white health coverage disparities remains an open question: Absolute disparities in coverage appear to have declined in expansion states, although exceptions have been reported. African American disparities in health access, treatment, or health outcomes showed little evidence of change for the general population.

**Conclusions:**

Future research addressing key weaknesses in existing research may help to uncover sources of continuing disparities and clarify the impact of future Medicaid expansion on African American health care disparities.

In the United States, stark health disparities can be observed between whites—the most advantaged group in terms of wealth and power—and African Americans—one of the most economically and socially disadvantaged groups. African Americans continue to fall significantly behind whites in 23 out of 29 indicators of health status, outcomes, and behaviors including life expectancy at birth and self-rated health, rates of diabetes, heart disease, asthma, and HIV, death rates from cancer, during infancy, and during and following pregnancy [1]. Driving these, in part, are disparities in healthcare access: higher rates of lacking a personal healthcare provider for regular care and lower vaccination and screening rates [1] and greater visitation of the emergency department for health care for ambulatory care sensitive conditions [2]. The ACA targeted lack of health insurance, a common barrier to healthcare access and utilization, which plays a key role in many of these disparities. Continuing health disparities between whites and African Americans decrease individual workforce participation, productivity, and generation of wealth and result in greater loss of life for African Americans. Disparities also result in considerable estimated direct ($136 billion) and indirect ($36.6 billion) public costs [3, 4].

The Affordable Care Act’s (ACA) commitment to reducing such seemingly intractable health disparities was emphatic. The text of the original bill (Pub. L. No. 111–148. 3–23–2010) contained 34 references to “disparities,” 28 references to either “discrimination” or “non-discrimination,” 33 instances using either the word “racial” or “race,” and 35 instances using either the word “ethnicity” or “ethnic” [5]. A key ACA instrument to increase health equality was expanding Medicaid eligibility to include all adults with incomes up to 138% of the Federal Poverty Line (FPL). Resulting increases in coverage were expected, in turn, to facilitate access to preventative care and treatment. Medicaid eligibility expansion was envisioned as a pathway to advancing health equity—an equal opportunity to be healthy [6].

Before expanded Medicaid under the ACA, and subsequently in non-expansion states, Medicaid eligibility was largely restricted to people deemed the “deserving poor” [7, 8]. This included pregnant women and children under six years of age, all poor school-aged children aged 6-18 if living in “deep poverty” (below half of the federal poverty level), parents with school aged children if living in “deep poverty,” children and adults with severe disabilities, and low-income older adults. Subsequently, only about 30% of poor single adults qualified for Medicaid coverage [9]. The ACA expanded Medicaid by eliminating previous eligibility requirements and by providing coverage for everyone with incomes below 138%. Due to the African American-white coverage, income and wealth gaps, expansion of Medicaid eligibility may be a powerful tool for reducing African American-white health disparities.

However, following a 2012 Supreme Court ruling, 19 states declined expanding Medicaid, and 12 states continue to decline it as of August 2021. Although denied expanded Medicaid coverage, persons with incomes between 100% and 400% FPL in non-expanding states qualified for subsidized purchase of private health insurance policies through ACA marketplaces. This possibility was denied persons with incomes below 100% FPL; disproportionately African Americans, such persons fell into a “coverage gap” [10].

African Americans’ over-representation in non-Medicaid eligibility expanding states may have limited achievement of the ACA’s disparity reduction goals for African Americans [10]. Given this variation in Medicaid expansion policies across states, how much Medicaid expansion furthered the ACA’s objective of closing African American-white disparities in healthcare coverage, access, treatment, and health outcomes is a key question to ask for evaluating the ACA’s disparity reduction aims.

## Understanding Medicaid expansions’ impact on disparities

Medicaid expansion focused on standardizing eligibility requirements, conferring eligibility on everyone with incomes below 138% FPL. Seeking to understand eligibility expansions’ impact on African American-white health disparities specifically, researchers capitalized on Medicaid expansion’s comprising a natural experiment with “treatment” (Medicaid expansion states) and control (non-expansion states) conditions. To attribute coverage, access, utilization, and health outcome disparity reductions to Medicaid expansion specifically, investigators must go on to explicitly compare (1) African Americans’ and whites’ coverage, access, utilization, and health outcome rates (2) before and after Medicaid expansion, in (3) expansion versus non-expansion states. If Medicaid expansion did indeed close African American-white health disparities, the differences-in-differences-in-differences (DDD) assessment should point to a significant interaction indicating that non-white versus white disparities declined (difference #1) following Medicaid expansion (difference #2) more in expansion states than in non-expansion states (difference #3). Individual and environmental controls are also needed to adjust for demographic and other differences, apart from race, which might bias comparisons and confound assessment of progress. Moreover, equity implies equal non-white/white proportions of coverage, access, treatment, and health outcomes given equivalent levels of need. Because pre-ACA rates of uninsurance, unmet health care need, and poor health outcomes were statistically relatively low, absolute and relative disparity metrics can differentially reflect change. For this reason, and because of substantive differences as to what “disparity” means, absolute and relative disparities should both be reported.

An equation making explicit these requirements is: Y_*ist*_ = β0 + β 1*Black_*i*_ + β 2*Expand_*s*_ + β 3*Post-ACA + β 4*(Black*Expand) + β 5*(Black*Post-ACA) + β 6*(Expand*Post ACA) + β 7*(Black*Expand*Post ACA) + ... + e_*ist*_ where the key parameter is the last, interacting African American status, Medicaid vs. non- expansion, and post-expansion time period. Our review’s concern is limited to the question of whether, nationwide, Medicaid expansion reduced disparities in Medicaid coverage and disparities in access and utilization of care and we select and interpret studies accordingly. We highlight the requirements outlined above to answer this key, but not exhaustive, question: as implemented nationwide in all of its facets, how much has Medicaid expansion reduced African American-white disparities? Though other methodological approaches—including single state case studies, regression discontinuity or interrupted times series analyses—can answer related and important question, this question is more fully and precisely answered with representative national data and a prioritization of the triple interaction.

Using these methodological standards as a conceptual framework for a review of this research the current study conducts a scoping review of the research to report on the state of knowledge about the impact of the Medicaid eligibility expansion on African American-white disparities in health coverage, access to healthcare, receipt of treatment, and health outcomes. To understand the whole impact of this policy, and the net effect of state variation in policy choices and implementation across the United States, we exclusively sample national studies. To identify the impacts of Medicaid expansion on African American-white disparities specifically, we apply analytic procedures described in the methodological description below, using the triple interaction approach as the benchmark for clearly addressing the central issue. The review assembles and interprets study findings, critiques methods, and identifies key questions for future study. It highlights areas in need of additional study to fully understand how much Medicaid expansion achieved African American-white disparity reduction and what lessons must be learned for further progress.

## Methods

A systematic search of the literature was conducted using the Preferred Reporting Items for Scoping Reviews/Meta-Analysis extension for Scoping Reviews (PRISMA-ScR) and evidence-based model utilization of PICO for framing questions a priori [11]. PICO components consist of Problem/Patient/Population, Intervention/Indicator, Comparison, Outcome, and (optional) Time element or Type of Study, which are essential in the formulated question and search criteria. The focus is the national population of non-disabled, non-elderly adults; the intervention of interest is Medicaid expansion; the comparator is Black and white racial identity; the outcomes of interest include health coverage, access, treatment, and outcomes or status; the time criteria requires that studies observe outcome pre- and post-Medicaid expansion; the Type of Study criteria requires that studies be quantitative. Thus, the focus of the scoping review was on investigations that were (1) nationwide, (2) assessed African American-white differences in coverage, access, treatment, and outcomes or status (3) before and after Medicaid expansion implementation (2014), and (4) compared Medicaid expansion and non-expansion states.

### Search strategy and study selection

A database search was conducted examining research reports from January 2014 through June 2021 to identify the sample of research studies to examine. This involved searching the following databases: CINAHL Complete, Health Source-Consumer Edition, Health Source: Nursing/Academic Edition, MEDLINE, APA PsychInfo, Psychology and Behavioral Sciences Collection, Social Work Abstracts. Abstracts were searched using the following terms: *African American or Black or African-American or Black American* AND *Medicaid expansion* AND *whites* AND disparit*. The search was conducted on July 1, 2021. Search results were narrowed to include only studies published in English. This yielded 47 articles. Of these articles, seven were removed (six duplicates, one dissertation). Full text review of the remaining 40 articles excluded 28 articles (19 non-national samples, five lacked pre- and post- ACA observations, three lacked a focus on Medicaid expansion, and one was non-empirical), leaving 12 articles remaining for further review. These studies were imported into a reference management system used to organize the literature.

A Kaiser Family Foundation (KFF) literature review on the effects of the Affordable Care Act’s (ACA) Medicaid expansion on health disparities was also closely examined for research reports [12, 13]. The KFF review examined published literature starting in January 2014 and ending in July 2020. KFF’s studies included all research on the impacts of Medicaid expansion for all race or ethnic groups for outcomes, including health coverage, healthcare access and utilization, and economic well-being for individuals and state governments. Abstracts from KFF’s 65 studies were screened for this review by four of the authors according to the criteria outlined above (national scope, assessed African American-white differences in coverage, access, treatment, and health outcomes before and after Medicaid expansion implementation, comparing Medicaid expansion and non-expansion states) resulting in 58 articles. Abstract screening eliminated 27 studies. Of the 31 remaining studies, 11 were eliminated after full-text review due to lack of national scope (*n* = 8), failure to identify Black-white disparities specifically (*n *= 2) and focus on non-target populations and outcomes (*n* = 1). This process yielded 20 studies from the KFF review, meeting the criteria. These studies were also imported into the reference management system.

**Fig. 1:**
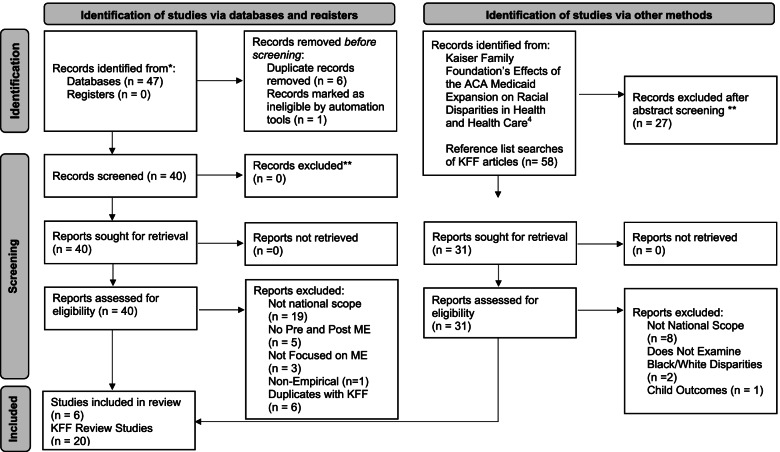
PRISMA-ScR 2020 flow diagram. Adapted from: Page MJ, McKenzie JE, Bossuyt PM, Boutron I, Hoffmann TC, Mulrow CD, et al. The PRISMA 2020 statement: an updated guideline for reporting systematic reviews. BMJ. 2021;372:n71. doi: 10.1136/bmj.n71. For more information, visit: http://www.prisma-statement.org/

The remaining 12 articles from the database search and screening were added to the 20 articles from the KFF sample. Within the 32 articles reported, six from the database search were duplicates of reports from the KFF sample and were removed. The review examined the remaining 26 articles or reports published from January 2014 through June 30, 2021, that use quantitative methods to investigate changes in health disparities between African American and white non-disabled, and non-elderly adults, before and after ACA Medicaid expansion, comparing states that did and did not expand Medicaid, using nationwide data. The PRISMA flow diagram (see Fig. 1) outlines the search strategy and screening results.

Using reference management software, three separate reviewers independently conducted databases searches and screened articles for inclusion based on inclusion criteria. Full text review was conducted by four members of the research team, and any conflicts about inclusion were resolved via discussion with the study's principal investigator (primary author). Interrater agreement was over 95%.

### Data extraction, analysis, and reporting

Critical review of the sample of studies focused on assessing the current state of knowledge about the impact of Medicaid expansion upon African American-white healthcare coverage, access, treatment, and health outcome disparities and questions remaining, given the strengths and limitations of each study. Using the triple difference research design as the standard to guide analysis, the data charting for each study included capturing the research aim, data sources, sample characteristics, covariates used, types of disparities measured, and key findings for each of the outcomes assessed. Outcomes of interest included health coverage, access to health care, and health care outcomes or health status. The analysis of research design specifically coded for how many of which differences were assessed, how disparities were measured (relative or absolute disparities), and what types of health coverage were assessed (public, private, or any-source health coverage). Findings were also coded for whether significance testing was conducted or reported for each difference.

## Results

Reporting formats vary, and information is presented to maximize comparability in Table 1. In this table, we organize studies in chronological order.

**Table 1:** Studies Examining Medicaid Expansion Impact on African American-white Health Care Coverage, Access, and Treatment, 2014-2021

Author (year)	Study Aim	Design/Differences Tested	Data Source	Sample Characteristics	Covariates	Type of Disparity	Un-Insurance Rates/Coverage	Treatment/Access to Care	Health Status/Outcomes
Menon, Patel, Karmakar, & Tipirneni (2021) [14]	Assessed differential impacts of ME on racial/ethnic and racial/ethnic-sex disparities among HIV testing	Difference-in-Difference (DD) and Triple Difference-in-Difference (DDD) Pre- & post-ACA ME & non-ME AA-white disparity	Behavioral Risk Factor Surveillance System (BRFSS), 2011-2018	Adults (ages 19-64), low-income (less than or equal to 138% FPL), non-pregnant, non-disabled	Age, sex, race, race-sex, percent FPL, education level, employment status	Absolute	N/A	ME associated with significant increase in reports of ever having HIV test; no significant changes in reporting HIV test in last year; no significant changes in AA-white disparities associated with ME in ever having HIV or having an HIV test in previous year	N/A
Johnson, Choi, & Herrera (2021) [15]	Compared changes in medication for opioid use disorder (MOUD) post-ACA to determine whether implementation was associated with increased MOUD for African American clients relative to white clients.	Descriptive and logistic regression analyses; associative analysis with interactions Pre- & post-ACA AA-white disparity	Substance Abuse and Mental Health Services Administration’s (SAMHSA) Treatment Episode Dataset-Admissions (TEDS-A), 2007-2018	White, African American, and Hispanic clients for opioid use disorder treatment, first annual episodes	Age groups, sex, education status, homelessness; client admission setting, heroin use, polysubstance use; geography (rural)	Absolute	N/A	No AA-white disparity changes associated with ME states; disparities increased in criminal justice-referred population in ME states; for Medicaid-covered population, disparities decreased; AA-white disparities in MOUD significantly reduced overall Significance of disparity in change difference between ME and non-ME states not tested	N/A
Ji, Castellino, Mertens, Zhao, Nogueira, Jemal, Yabroff, & Han (2021) [16]	Examined association of ME with insurance coverage at diagnosis among young adults newly diagnosed with cancer	Difference-in-Difference (DD); used linear probability models Pre- & post-ACA ME & non-ME Examined both public and private coverage	National Cancer Database (NCDB), 2011-2016	Young adults (ages 18-39) diagnosed with a first primary cancer between January 1, 2011 and December 31, 2016 in U.S.	Sex, age, group, self-reported race/ethnicity, urban or rural residence, median household income	Absolute	Not examined by race/ethnicity	Stage 1 diagnosis increased more in ME states than non-ME states for white patients and AA patients; increase larger but less significant for AA patients AA-white disparity or changes in disparities associated with ME not tested for significance	N/A
Le Blanc, Heller, Friedrich, Lannin, & Park (2020) [17]	Reviewed association of ME with breast cancer state at diagnosis and disparities associated with insurance status, age, and race/ethnicity	Retrospective cohort analysis (XW analysis used, with 1-sided *p* < 0.05; Mann-Whitney U test used to assess significance of state income and employment data with 1-sided *p* < 0.05) Pre- & post-ACA Distinguished between Medicaid coverage and un-insurance	National Cancer Database (NCDB) public benchmark reports via the American College of Surgeons, 2007-2016	Patients with primary breast cancer diagnosed between 2007 and 2016 who were uninsured or had Medicaid, private insurance, or Medicare, and whose race/ethnicity, age, state of residence, and American Commission Joint Cancer summary were recorded	Race, insurance status (uninsured and Medicaid), breast cancer stage (early or late)	Absolute	ACA implementation associated with reduced number of uninsured white and AA patients in ME and non-ME states; reductions greater for all racial groups Significance of AA-white disparities not tested; differences between ME and non-ME states not tested for significance	ACA associated with increased rates of cancer diagnosis at earlier stages for AA and white patients in ME states, but not in non-ME states; incidence of advanced disease in AA patients decreased in ME states, and remained approximately the same in non-ME states Significance of AA-white disparities not tested; difference between ME and non-ME states not tested for significance	N/A
Baumgartner, Collins, Radley, & Hayes (2020) [18]	Examined degree to which racial/ethnic disparities have narrowed post-ACA	Difference-in-Difference (DD) Pre- & post-ACA AA-white disparity Did not distinguish between private and public insurance	American Community Survey Public Use Microdata Sample (ACS PUMS), 2013-2018 Behavioral Risk Factor Surveillance System (BRFSS), 2013-2018	Adults (ages 18-64); white, African American, Hispanic	None reported	Absolute	AA adults living in ME states now less likely to be uninsured than white adults in non-ME states No statistics of significance reported	Unmet need due to cost decreased for AA and white; AA-white differences in cost-related access problems have narrowed in ME and non-ME states No statistics of significance reported Having usual source of care increased for white and for AA; AA adults in ME states now almost as likely as white to have usual source of care No statistics of significance reported	N/A
Buchmueller & Levy (2020) [19]	Considered how the ACA’s insurance coverage expansions have affected racial/ethnic disparities related to access to care	Estimated difference between groups; presented unadjusted mean outcomes and results that controlled for individual characteristics Pre- & post-ACA AA-white disparity Did not distinguish between private and public insurance	Behavioral Risk Factor Surveillance System (BRFSS), 2008-2017	Adults (ages 19-64); ~ 400,000 adults each year	Age, education, employment status, sex, marital status, and interaction between sex and marital status	Absolute	National AA-white un-insurance disparity decreased after ACA implementation Difference in AA-white disparity reductions between ME and non-ME states not tested for statistical significance	National AA-white foregone care due to cost disparity decreased after ACA implementation Difference in AA-white disparity reductions between ME and non-ME states not tested for statistical significance	N/A
Artiga, Orgera, & Damico (2020) [20]	Examined how health coverage by race/ethnicity has changed post-ACA	Difference-in-Difference (DD), stratified by race/ethnicity Pre- & post-ACA ME & non-ME Did not distinguish between private and public insurance	American Community Survey (ACS), 2010-2018	Non-elderly population of whites, African Americans, Hispanics, & Alaska Natives (ages 0-64)	National coverage time trends	Absolute	Un-insurance rates for whites and AA decreased in non-ME and ME states, 2010-2018 DDD effect not tested for significance for AA-white disparity change	N/A	N/A
Breslau, Han, Lai, & Yu (2020) [21]	Examined impact of ME on use of 4 types of mental health services in nationally representative samples of low-income individuals during first 2 years following implementation	Difference-in-Difference (DD); used survey design-adjusted linear regression models; binary modeled by logistic DD model; continuous modeled by linear DD regression on subsample of service users; impacts of ME within racial/ethnic groups examined by extending original DD models to DDD models Pre- & post-ACA ME & non-ME	Medical Expenditure Panel Survey (MEPS), 2007-2015	Adults (ages 18 and older) with incomes at or below Medicaid eligibility under expansion rules (138% FPL)	Age, sex, marital status, race/ethnicity, K-6 score category, education level	Absolute	N/A	Change in use of outpatient mental health visits significant among whites and Hispanics, but not AA; no significant changes observed in number of mental health-related hospital stays, emergency department visits, or prescription refills Significance in changes in AA-white disparities due to ME implementation not tested for any outcomes	N/A
Wiggins et al. (2020) [22]	Determined association between ME and infant mortality rates (IMR) in U.S.	Difference-in-Difference (DD); multiple linear regression models using DD estimation and Huber-White robust standard errors Pre- & post-ACA ME & non-ME	CDC’s Wide-ranging Online Data for Epidemiologic Research (WONDER), 2019-2017	State-level aggregate data on U.S. IMR and population count	Sex, race/ethnicity	Absolute	N/A	N/A	No association between ME states and change in national IMR, 2010-2017; ME associated with reduction in IMR among Hispanics; ME not associated with IMR reduction in ME states relative to non-ME states for whites and AA Significance of AA-white disparity changes related to ME or ACA not tested
Barrington, Simmot, Calo, Cohn, Cosgrove, & Felix (2020) [23]	Determined associations between ME adoption and changes in insurance status, early-stage diagnosis, and cancer survival among women with endometrial carcinoma	Difference-in-Difference (DD); overall survival was fit with Cox proportional hazards models; logistical regression compared epidemiological, hospital, tumor, and treatment characteristics of women according to dichotomized ME status Pre- & post-ACA ME & non-ME Examined a variety of insurance sources	National Cancer Database, Participant User Files (PUF), 2004-2015	Patients diagnosed with invasive endometrial carcinoma (ages 40-64)	Facility location, ME category of patient ZIP code; year (grouped into pairs); age, race, comorbities, hospital type, hospital location, rurality, educational attainment, household income, year of diagnosis	Absolute	Statistically significant improvement of changes in percent insured associated with ME implementation in ME states among white, but not African American. DDD effect not tested for significance for AA-white disparity change	No significant changes observed in early-stage diagnosis associated with ME for any race. DDD effect not tested for significance for AA-white disparity change	Significant increases in overall survival rates observed to be associated with ME for whites; no significant changes in survival rates observed for AA women. DDD effect not tested for significance for AA-white disparity change
Eliason, 2020 [24]	Examined the effect of Medicaid expansion under the Affordable Care Act on state-level maternal mortality ratios in the United States.	Difference-in-Difference (DD) Pre- & post-ACA ME & non-ME Stratified by race	Underlying Cause of Death 2006–2017 data files from the National Center for Health Statistics; Centers for Disease Control and Prevention, National Center for Health Statistics Natality data files, CDC WONDER Online Database	50 states and DC, from 2006 to 2017, for a total of 612 state-year observations	State-wide pregnancy checkbox adoption and state-level women’s unemployment ratio	Absolute	N/A	N/A	ME was significantly associated with lower maternal mortality; ME effects were concentrated among non- Hispanic Black mothers. AA-white disparity or changes in disparities associated with ME not tested for significance
Brown, Moore, Felix, Stewart, & Tilford (2020) [25]	Identified association of ME with changes in county-level geographic variation in rates of low birthweight and preterm births, overall stratified by race/ethnicity	Compared changes in coefficient of variation and ratio of 80^th^ to 20^th^ percentiles using bootstrap samples (*n* = 1,000) of counties drawn for all births and for white, African American, and Hispanic births, separately Pre- & post-ACA ME & non-ME AA-white disparity	National Center for Health Statistics (NCHS) Vital Statistics Birth Data Files, 2011-2016	County-level rates of low birthweight and preterm birth outcomes; 3,145 counties in contiguous U.S., excluding counties in U.S. territories; sample counties (*n* = 372) in 6 contiguous states that expanded Medicaid after January 1, 2014, and 9 independent cities, leaving 2,728 counties; county-level rates in included counties among 19,454,243 singleton births to women (ages 19 and older at time of birth)	None reported	Absolute	N/A	N/A	County-level variation for low birthweight and preterm births among all racial/ethnic categories declined in ME states; in non-ME states, geographic variation reduced for both outcomes among Hispanic births and low birthweight white births, but increased in both outcomes among AA births Significance in changes in AA-white disparities not tested for either outcome
Glance, Thirukumaran, Shippey, Lustik, & Dick (2020) [26]	Determined whether ME was associated with reduction in revascularization disparities in patients with acute myocardial infarction	Retrospective analysis study; comparative interrupted time series analysis Pre- & post-ACA ME & non-ME AA-white disparity Did not distinguish between private and public insurance	Vizient Clinical Database/Resource (CDB/RM), 2010-2018	White and African American patients (ages 18-64) hospitalized with ST-segment elevation (STEMI) or non-ST-segment elevation acute myocardial infarction (NSTEMI) after ME	Patient characteristics, pre-ACA temporal trends, hospital effects	Absolute	Among patients with STEMI and NSTEMI, AA-white un-insurance rate disparity reductions in ME vs. non-ME states before and after ACA (DDD) is significant at *p* < 0.001.	N/A	Differences in AA-white revascularization rates for patients with STEMI decreased by 2.09 percentage points per year in ME vs. non-ME states; 7.24 percentage point increase for AA patients hospitalized with STEMI in non-ME states
Semprini & Olopade (2020) [27]	Evaluated impact of ME on disparity between African American-white breast cancer mortality rates	Difference-in-Difference (DD) fixed effects regression model with AA-white mortality ratio as outcome Pre- & post-ACA ME & non-ME AA-white disparity	CDC All-Cause Mortality Database, 2012-2016	State-level breast cancer mortality data; no additional inclusion or exclusion criteria reported	Age	Absolute	N/A	N/A	ME did not lower disparity in breast cancer mortality; AA-white mortality ratio increased in ME states for all Medicaid-eligible age groups with significant effects in younger age groups
Chaudry, Jackson, & Glied (2019) [28]	Determined the extent to which the ACA has reduced disparities in insurance coverage among different racial/ethnic groups	Difference-in-Difference (DD) Pre- & post-ACA; estimates adjusted Examined public, private, and no coverage separately	American Community Survey (ACS), 2013-2017	Adults (ages 19-64); white, African American, Hispanic (any race); grouped by income relative to federal poverty guidelines;	Stratified by income level	Absolute	Among all income levels, white un-insurance rates declined in non-ME and ME states; AA and non-Hispanic un-insurance rates decreased in non-ME and ME states Rates unadjusted and no significance testing conducted	N/A	N/A
Singh & Wilk (2019) [29]	Examined changes in access to primary care, measured by insurance status, having usual source of care, and delaying care due to cost, following ACA ME	Difference-in-Difference (DD); logistic regression models Pre- & post-ACA ME & non-ME AA-white disparity Did not distinguish between private and public insurance	Behavioral Risk Factor Surveillance System (BRFSS), 2011-2016	Adults (ages 25-64); white, African American, and Hispanic; other non-Hispanic adults (ages 25-64) with incomes below 100% FPL	Age, education status, marital status, gender, self-rated health status; race/ethnicity, income	Absolute	No significant AA-white disparity changes in insurance rates due to ME	No significant AA-white disparity changes due to ME status in unmet need due to cost or having a usual source of care	N/A
Lipton, Decker, & Sommers (2019) [30]	Examined changes related to racial/ethnic disparities in health insurance coverage and access to care after implementation of dependent coverage provision and full ACA limitation in 2014, respectively, separate from preexisting trends	Interrupted time series approach with 2 distinct intervention periods: October 2010 to December 2013 and January 2014 to December 2014 Pre- & post-ACA ME & non-ME AA-white disparity Examined public, private, and any coverage	National Health Interview Survey (NHIS), 2000-2014	48,358 young adults (ages 19-25)	Age, sex, marital status, education, employment status, family income, region of residence; models included linear quarterly trend to control for trends in each outcome prior to ACA implementation	Absolute	ME associated with significantly greater increases in rates of having health coverage and having Medicaid coverage compared to gains for whites; rates of reporting any type of health insurance increased at significantly greater rates than for whites in ME states	ME associated with significantly greater increases in reporting a usual source of health care for AA compared to gains for whites; cumulative changes for ACA associated with significantly greater increases in reporting at least one doctor’s visit for AA compared to gains for whites	N/A
Crocker, Zeymo, McDermott, Xiao, Watson, DeLeire, Shara, Chan, & A-Refaie (2019) [31]	Examined impact of ME on utilization of cancer surgery for uninsured overall, low-income persons, and racial minorities	Poisson interrupted time series (ITS) analysis Pre- & post-ACA ME & non-ME AA-white disparity Examined private and public insurance separately	Merged data from State Inpatient Database, American Hospital Association, and Area Resource File, 2012-2015	81,000 patients (ages 18-64) who underwent cancer surgery	Adjusting for age, sex, comorbidity score, population-level and provider-level characteristics; quarter of discharge; year of admission; payer type	Absolute	N/A	Medicaid and uninsured population in ME states substantially increased utilization relative to non-ME states in 2014 DDD effect not significant for AA-white change and cancer surgery utilization	N/A
Wehby & Lyu (2018) [32]	Examined ACA ME effects on Medicaid take-up and private coverage and coverage disparities by age, race/ethnicity, and gender	Stratified Difference-in-Difference (DD) regression with state fixed effects; excluded 14 states that had partial or full expansions prior to 2014 Pre- & post-ACA ME & non-ME Examined Medicaid coverage, uninsured, individually purchased, employer-sponsored coverage, any private coverage	American Community Survey, 2011-2015	3,137,989 low-educated (high school or less) adults (ages 19-64); did not select sample based on household income or poverty level because income is potentially endogenous to insurance	National coverage time trends stratified by age group, stratified by race/ethnicity and gender	Absolute	Slight change in coverage disparities by race/ethnicity DDD effect not tested for AA-white disparity change	N/A	N/A
Han, Yabroff, War, Brawley, Jemal (2018) [33]	Examined changes in percent uninsured and percent reporting care unaffordability, pre- & post-ACA ME	Difference-in-Difference (DD) Pre- & post-ACA ME & non-ME Did not distinguish between private and public insurance	Behavioral Risk Factor Surveillance System (BRFSS), 2011-2017	118,631 cancer survivors (ages 18-64) with no known sex	Gender, age, race/ethnicity, household income, education, employment status, marital status, number of comorbid conditions	Absolute	Percent uninsured and care affordability decreased in all racial groups; disparities between white and Hispanic survivors persisted; greater reductions in un-insurance rates for AA in ME states (not statistically significant) DDD effect not tested for significance for AA-white disparity change	Greater reductions in rates of care affordability reports for AA in ME states (not statistically significant); no significant findings for ME impact on AA-white disparities in unmet need due to cost DDD effect not tested for significance for AA-white disparity change	N/A
Lee & Porell (2018) [34]	Estimated impacts of ACA ME on racial/ethnic disparities in insurance coverage, access to care, and health status	Difference-in-Difference (DDD) model specification with treatment and comparison groups; linear probability and regression models Pre- & post-ACA ME & non-ME AA-white disparity Did not distinguish between private and public insurance	Behavioral Risk Factor Surveillance System, 2011-2016	Non-pregnant childless adults (ages 19-64) residing in U.S. state or D.C. with incomes less than 100% FPL	Age, gender, race, marital status, education, employment, chronic disease status, tobacco use; state-year variables including number of hospital beds and physicians per 1,000 population, unemployment rate, per capita income, racial/ethnic composition, Senate voting records	Absolute	DDD effect not significant for AA-white disparity change in un-insurance	No significant findings for ME impact on AA-white disparities in unmet needs due to cost, having a usual source of care, or having annual wellness exam	No significant findings for ME impact on AA-white disparities in reported fair or poor physical health days, number of poor mental health days, and days with health-related activity limitation
Yue, Rasmussen, & Ponce (2018) [35]	Examined impacts of ME on health insurance coverage, having personal doctor(s), being unable to see doctors because of cost, and receiving a flu shot; tested racial/ethnic differential impacts	Quasi-experimental design with Difference-in-Difference (DDD) analyses; multiple imputations and survey weights used; excluded 14 states that had partial or full ME prior to 2014 Pre- & post-ACA ME & non-ME AA-white disparity Did not distinguish between private and public insurance	Behavioral Risk Factor Surveillance System (BRFSS); State Physicans Workforce Data Book; Bureau of Labor Statistics, 2013-2015	Adults (ages 18 or older), low-income, non-elderly based on household income and family size; 18,408 observations in non-ME group and 16,964 in ME group	Age, general health, annual household income, race, education level, employment status, language, number of children in household, number of adults in household	Absolute	DDD effect not significant for AA-white disparity changes in rates of any-health coverage	No significant findings for ME impact on AA-white disparities due to cost, having usual source of care or personal doctor, or having flu shot	N/A
Hayes, Riley, Radley, & McCarthy (2017) [36]	Investigated effects of ME on access to health care across three racial/ethnic groups Did not distinguish between private and public insurance	Stratified analysis; calculated and compared national averages for each indicator pre- & post-ACA, comparing ME and non-ME states No statistical tests performed	American Community Survey (ACS), 2013 & 2015 Behavioral Risk Factor Surveillance System (BRFSS), 2013 & 2015	Uninsured adults (ages 19-64); adults (ages and older) who identified unmet heath care need do to cost and lacks usual source of care	None reported	Absolute	AA-white absolute disparity in un-insurance rates decreased in non-ME and ME states No statistics of significance reported	AA-white absolute disparity in rates of unmet health care need due to costs decreased in non-ME and ME states No statistics of significance reported AA-white absolute disparity in rates of lacking a usual source of care decreased in non-ME and ME states No statistics of significance reported	N/A
Flores & Vargas (2017) [37]	Tested whether ME predicted change in ethnoracial disparities with health insurance coverage at the county level	Fixed-effect regression models Pre- & post-ACA ME & non-ME AA-white disparity Did not distinguish between private and public insurance	American Community Survey, 2012-2014	U.S. counties with large enough minority population to conduct meaningful cross-race analyses; not a nationally representative study of U.S. counties	County-level immigration-related policy (20112-2014 from National Conference of State Legislatures); state-level racial prejudice (Google); county obesity rates (CDC); annual county median age and ethnoracial composition; baseline insurance coverage levels; annual county unemployment rate	Absolute	Gaps in county any-insurance coverage rates between AA and whites decreased, 2012-2014 DDD effect not significant for AA-white disparity change	N/A	N/A
Buchmueller, Levinson, Levy, & Wolfe (2016) [38]	Examined ACA and ME effect on rates of un-insurance, public health coverage, and private health coverage by racial/ethnic groups	Difference-in-Difference (DD) stratified by income group and state ME status Pre- & post-ACA ME & non-ME AA-white disparity Distinguished between private and public insurance rates	American Community Survey, 2008-2014	Adults (ages 19-64), white, African Americans, and Hispanics (any race)	Stratified analysis by income group and state ME status; did not control for sociodemographic or health status factors	Absolute	AA-white coverage gap decreased for both public and private insurance; greater gains for AA adults in non-ME states; greater gains for whites in ME states; AA without health insurance decreased in ME and non-ME states DDD effect not significant for AA-white disparity change in un-insurance rate	N/A	N/A
McMorrow, Long, Kenney, & Anderson (2015) [39]	Examined ME impacts on absolute & relative disparity changes in un-insurance rates for African Americans and whites	Difference-in-Difference (DD) Pre- & post-ACA AA-white disparity Did not distinguish between private and public insurance	National Health Interview Survey (NHIS), 2012-2014	Adults (ages 18-64); white, AA, Hispanic	Age; sex; did not control for sociodemographic or health status factors	Absolute & Relative	Absolute disparity for AA uninsured adults in ME and non-ME states decreased, 2013-2014 DDD effect not tested for significant differences in AA-white disparity in ME vs. non-ME states	N/A	N/A

### Data sources

Investigators reported national findings for the general U.S. population or persons with an identified illness. The former used nationally representative surveys providing information on insurance coverage—usually any coverage or reduction in un-insurance—and indicators of healthcare access and utilization. The latter used heath records, registries, and other databases tracking persons with the illness of concern and providing information on coverage and treatment (see Table 1).

### Difference in difference study designs

Three studies either assessed a single difference excluding the triple interaction or used unadjusted estimates [17, 28, 36]. Twelve studies tested double differences. Of these, four studies tested differences in outcomes before and after the ACA and between African Americans and whites but failed to test differences between expansion and non-expansion states [15, 18, 19, 39]. Eight studies tested differences in outcomes before and after the ACA and between expansion and non-expansion states but failed to test differences between African Americans’ and whites’ outcomes [16, 20–24, 32, 33]. Eleven studies tested all three differences: before and after the ACA implementation, between Medicaid expansion and non-expansion states, and between African Americans and whites [14, 25–27, 29–31, 34, 35, 37, 38].

### Study results: Changes in coverage disparities

The research documents significant gains in coverage associated with the ACA, but it clarifies surprisingly little about Medicaid eligibility expansion’s impact on African American and white racial disparities in Medicaid coverage. Un-insurance is the most commonly examined outcome variable (17 studies), but only eight of these studies specify public or private health coverage outcomes [16, 17, 23, 28, 30–32, 38]. Findings for coverage disparity reduction are mixed. Percentage point reductions in un-insurance disparities were shown under Medicaid expansion [19, 34, 36, 38, 40], but several studies reported that disparity reductions were not statistically significant [29, 34, 35, 37, 38]. Several failed to report statistical testing of disparity reduction itself [17, 19, 20, 23, 28, 32, 33, 36, 39]. Three studies documented reversed expectations, showing greater coverage gains for African Americans than whites in non-Medicaid expansion states [30, 38, 39].

In studies focusing on populations with specific illnesses, one study found Medicaid expansion to be associated with African American-white disparity reduction in coverage [26]. Other studies focusing on patients with specific health conditions found no significant disparity reduction in coverage for patients with specific conditions or failed to test for significant changes in disparities [17, 23, 41].

### Study results: Changes in access and treatment disparities

Medicaid eligibility expansion disparity reduction in access and treatment were examined only in 14 out of the 26 studies, and findings were mostly negative. While one research team reported that disparity reduction was greater in expansion states for young adults [30], the majority of studies reported no statistically significant effects for African American-white disparities [14, 18, 29, 31, 34, 35, 41] or failed to report significance testing [15, 16, 18, 19, 21, 23, 36]. Though not studied widely in the general population—only seven general population studies examined outcomes beyond coverage—disparities in indicators of healthcare access (usual source of care, having a personal doctor, delaying care due to cost) and treatment (having a wellness exam, flu shot) appear to be unchanged by Medicaid expansion [18, 19, 21, 29, 34–36]. In studies focusing on populations with specific illnesses, access to treatment for specific conditions either showed no significant disparity reductions due to Medicaid expansion [31] or failed to test the significance in either disparity changes or differences between Medicaid expansion and non-expansion states [15–17, 23, 41].

### Study results: Changes in health status or outcome disparities

Only seven of the 26 studies examined African American disparity reductions in health outcomes [22–27, 34]. One study found that expansion was not associated with significant changes in self-reported health status, number of poor physical or mental health days, or days with health-related activity limitations [34]. County-level variation rates of low infant birth weight or preterm births reduced for African Americans in expansion states and increased in non-expansion states—but the size or significance of the racial disparities or changes in them due to expansion was not tested [25]. No significant changes in infant mortality rates were observed in either expansion or non-expansion states for whites or African Americans [22]. However, in a study examining changes in maternal mortality, expansion was significantly associated with reductions in maternal mortality rates. Reductions in Medicaid expansion states were largest for Black mothers, but the size of Black-white disparities before or after expansions or the significance of any changes in disparities were not measured or tested [24]. No significant disparity reductions were found in survival rates in patients with specific life-threatening health conditions [23, 26, 27].

## Discussion

This review indicates that African American disparities in health access, treatment, or health outcomes—with the important exception of maternal mortality rates—remain largely unchanged by Medicaid expansion. However, whether Medicaid eligibility expansion reduced African American-white health coverage disparities remains an open question: Absolute disparities in coverage appear to have declined in expansion states, although exceptions have been reported. Future research addressing key weaknesses or oversights in existing research may help to uncover sources of continuing disparities and clarify the impact of Medicaid expansion on changes in health coverage disparities.

### Improving research precision and rigor

Improved research efforts can clarify the answer to this question—and identify structural sources of continuing disparities—by more carefully targeting Medicaid eligibility expansion as a source of disparity reduction and accounting for, or specifically examining, the role of variation in broader ACA-related health system changes. Further studies should examine changes in relative health disparities as well as absolute health disparities and must examine disparity changes for African Americans separately from disparity changes for other racial or ethnic groups. Deeper investigations of African American-white disparity reductions in healthcare access, treatment, and health outcomes—which appear to be relatively unchanged by Medicaid expansion—should consider community and provider-level treatment contexts that may impact African Americans especially and have sometimes been impacted by the ACA’s health reforms.

#### Testing the triple interaction

To test disparity reduction directly, studies need to document significant reductions in the differences between 1) African American and whites’ coverage, access, utilization, and health outcome rates, 2) before and after Medicaid expansion, 3) in expansion versus non-expansion states. Only 11 out of 26 studies tested for the significance of all three differences. Of these studies, no study examining the general population found significant disparity reductions in health coverage, treatment, access, or health outcomes associated with Medicaid expansion. However, coverage disparity reductions were found for young adults and patients with acute myocardial infarctions. Less than half of the sample studies used a full triple difference analysis, and to overcome present uncertainty, investigators must routinely comply with this requirement.

We discuss requirements for the DDD design because in studies qualifying for our review, investigators uniformly chose such designs. Other types of Medicaid expansion studies and analytic approaches are desirable for many purposes. Studies of single states or groups of states that do not employ DDD designs or highlight the triple interaction we emphasize, can provided insight into key questions about Medicaid expansion disparities, and our framework should not be considered to downplay potential contribution from approaches falling outside of our framework. For example, using interrupted time series to study surgical cancer care in New York state, investigators found that Medicaid expansion increased disparities in access to high quality hospitals [42]. This important finding alerts policy makers in a large state to a key issue and provides a foundation for follow-up studies in comparable states. Medicaid expansion encompasses several system changes beyond relaxed eligibility whose contribution to disparity reduction warrants study and which may be achieved through other methods, including single state case study, regression discontinuity or interrupted time series designs.

#### Absolute versus relative disparities

Existing examinations of Medicaid expansion impacts on health disparities almost exclusively report gains in absolute disparities—Black-White percentage point increases or decreases in coverage, access, and treatment. Yet relative disparities—African Americans’ proportion of coverage, access, and treatment relative to Whites’ proportion—represent an alternative and widely accepted point of view on disparities [43, 44]. The two indicators need not agree and can even give opposite readings [44]. For example, the magnitude of absolute and relative Black–White disparities in infant mortality rates in the US changed in opposite directions during the twentieth century [45].

There is reason to believe that Medicaid expansion evidence has not escaped oversimplified conclusions from an almost exclusive reliance on absolute disparities. In this review, only one study was identified that examined relative disparities at all by reporting both absolute and relative disparities [39]. The investigators found that absolute disparities significantly decreased in expansion states--but that they decreased also in non-expansion states. However, completely upending expectations, relative disparities were *not* significantly reduced in expansion states--but they were significantly reduced in non-expansion states. Apparently, only in non-expansion states was absolute disparity improvement great enough to move the relative disparity dial. For a full understanding of the impact of Medicaid expansion on African American-white disparities, investigators should report relative as well as absolute disparities and carefully interpret any differences that might arise.

#### Disaggregating Medicaid expansion from other ACA elements

The ACA ushered in many innovations apart from the Medicaid eligibility expansion. The Medicaid application process was streamlined as online filing options increased and verification and certification procedures capitalized on new technologies [46]. Individuals with incomes between 100% and 400% FPL became eligible for “Premium Tax Credits” on a sliding scale to purchase private, non-group coverage through state or federally-operated healthcare exchanges [47], and persons with incomes between 100% to 250% FPL became eligible for cost-sharing subsidies. Gains were concentrated among those with incomes between 138-250% of the FPL—those who were eligible for the ACA’s cost-sharing reductions and among whom African Americans are also over-represented [48, 49]. In non-expansion states, premium tax credits and subsidies could offset denial of access to expanded Medicaid for persons with incomes above 100% FPL.

Marketplaces, which informed inquiring persons about Medicaid eligibility, actively sought enrollees through vigorous outreach efforts. Community targeted advertising raised awareness, and marketplaces provided individual counseling on eligibility and options, sometimes facilitated by culturally sensitive enrollment assistors [50]. Safety net hospitals faced new incentives to avoid hospital readmission and reduce lengths of stay by shifting newly eligible patients to Medicaid-funded outpatient care [50]. Funding was increased for new Federally Qualified Health Centers, which disproportionately support African Americans through targeting services for the poor [51]. These and other developments promised to reduce barriers to coverage and access for non-white, low-income adults—lessening healthcare disparities throughout the United States as many previously eligible people become aware of Medicaid eligibility and enrolled (“woodwork effect”) [52]. New research must examine the impacts of ACA policy elements on disparities in specific types of health insurance coverage rather than on the all-inclusive “un-insurance.”

### Advancing knowledge: Beyond Medicaid expansion’s eligibility requirements

Additional advances in research should examine variation in state implementation of Medicaid expansion. This includes attention to the role the Section 1115 Medicaid waivers have played in expanding Medicaid eligibility—both before and after the ACA’s implementation—and the extent to which changes in health coverage disparities are attributable to enhanced awareness of health coverage possibilities resulting from vigorous outreach and health coverage enrollment efforts in both expansion and non-expansion states.

#### 1115 Medicaid Waivers

Medicaid 1115 waivers were issued to 14 states between 2004 and 2012 for early Medicaid expansion, and, in some states, early expansion significantly affected coverage rates [53]. Two studies excluded these states from consideration [32, 35], but others failed to account for the possible pre-ACA reduction in coverage increase and disparity. Investigators may have underestimated ACA expansion’s impact on disparities by neglecting early expansion. Medicaid waivers played a dual role in Medicaid eligibility expansion.

In addition to the 1115 waivers approved prior to the ACA Medicaid expansion, four states (Arizona, Arkansas, Iowa, and Michigan) accepted Medicaid expansion, but received approval to expand Medicaid in ways not otherwise allowed under federal laws through Section 1115 Waivers. In the years following the ACA Medicaid eligibility expansion, several initially rejecting states expanded Medicaid eligibility through Section 1115 waivers (e.g., Indiana in 2015, Montana in 2016, and Utah in 2020). These states used these waivers to customize eligibility standards to accommodate better ideological and fiscal reservations [54]. Some states expanded Medicaid with restrictions—requiring premium payment to begin coverage, using health savings accounts, tying healthy activities to waived premiums (e.g., New Mexico, Texas, Georgia), or including work requirements (e.g., Indiana, Kentucky). These are complex to implement and present grave administrative challenges [55, 56], reducing uptake of Medicaid coverage [57]. Arkansas’ coverage gains did not differ in gains from traditional Medicaid expansion [58], but Arkansas’ addition of work requirements in June 2018 resulted in thousands losing coverage—reportedly due to administrative complexity [59]. African Americans have experienced race-related aversive experiences with bureaucratic programs [60], and waiver-imposed barriers may deter African Americans especially. More research is needed to identify the impact of waivers on disparities. This knowledge is critical to informing future approvals for state maneuvers to expand Medicaid conditionally or partially through these policies. Currently, 63 waivers have been approved across 45 states, and 28 applications in 22 states are currently pending decisions from the Centers for Medicaid and Medicare Services (CMS) [61].

#### Outreach and enrollment assistance

Disparity reduction in non-expansion states points to the possibility that some states reduced enrollment barriers for African Americans especially. Advertising, enrollment assistance, and greater enrollment incentives for FQHCs and safety net hospitals to maximize enrollment likely increased Medicaid uptake. Conceivably, previously uninsured African Americans who were eligible for Medicaid prior to Medicaid expansion disproportionately responded to ACA messages about coverage possibilities, were less deterred by burdensome enrollment procedures due to streamlining efforts under the ACA or were disproportionately gaining enrollment through newly available Federally Qualified Health Centers or in safety-net hospitals as they encouraged covered outpatient care.

### Populations with Chronic or Critical Conditions

Among eight studies focusing on populations with specific illnesses, one study found Medicaid expansion to be associated with African American-white disparity reduction in coverage [26]. However, none of these studies report significant reductions in disparities in access to treatment, survival rates, or health outcomes. Coverage disparity reductions in populations with critical or chronic conditions, and associated changes in access to care, must be considered considering the presence of strong incentives to find insurance coverage for costly medical procedures. Providers are motivated to facilitate enrollment to avoid the burden of uncompensated care—the very “adverse selection” that concerns insurers and necessitated the ACA’s requirement that persons with pre-existing conditions not be denied coverage [62]. Opportunities for gaining coverage are likely more available in expansion states, and thus coverage disparity reductions observed under strong incentives to enroll must be understood on their own terms and may not be generalized to the wider population.

### Access, Treatment, and Health Outcomes

This review also highlights that there is limited evidence supporting the expectation that disparity reductions in coverage translated into disparity reductions in access, utilization, or health outcomes. Refinements are needed to determine better whether such reductions occurred and how. Studies assessing access and treatment utilization should consider other non-cost-related barriers to healthcare access—including barriers that may impact African Americans especially. Size and location of provider supply, program outreach and cultural responsiveness, and other determinants of receiving care may be relevant. An expansion of Community Health Centers funded by an ACA-created trust fund, where African Americans disproportionately are treated, is particularly ripe for study as an ACA-related trigger for change in provider supply. Focusing directly on access and treatment disparities is indicated, taking us beyond inconclusive findings from present approaches measuring only the onset of the ACA and its immediate impacts on coverage disparities.

Examinations of the ACA’s impact on disparities in health outcomes and health status—which may result from higher health insurance rates but will likely take longer to emerge—should also be examined in the coming decade. Due to the impact of a wide range of social determinants upon health—and the disproportionate exposure of African Americans to determinants that negatively impact health status and health outcomes [63]—the impacts of the ACA on health outcomes will be complex to untangle and likely more difficult to detect.

## Conclusion

Stressing non-discrimination and promoting cultural sensitivity [5, 50], the ACA sought to reduce, if not eliminate, racial and ethnic disparities in insurance coverage, access, treatment, and health outcomes. The ACA introduced a suite of disparity-sensitive policy tools to achieve these aims. Preliminary findings regarding African American disparity reductions in healthcare access, receipt of treatment, or health outcomes are discouraging, and structural sources of continued disparities call for deeper investigations of ongoing barriers to care.

Global improvement appears to have occurred in health coverage disparities, and these are associated with the onset of the ACA. Disentangling the role of a prominent instrument for disparity reduction—Medicaid expansion—remains elusive and considerable room persists for additional disparity reduction. For gains that have been achieved in health coverage disparity reduction, it is unclear how much gains in coverage were due to expanded eligibility for Medicaid and how much was due to energetic efforts to encourage take-up.

The ACA is built upon long-existing health care policy [64] and has become intricately incorporated into the U.S. health care system [65]. Incremental policymaking theories indicate that future policing health will build upon the policy lever established under this policy [66], as is exemplified by the recently enacted American Rescue Plan Act, which extended and increased the marketplace subsidies and increased state incentives to participate in Medicaid eligibility expansion. Thus, identifying policies and actions under the ACA that failed to adequately close gaps in health coverage and treatment for African Americans and isolating the most potent ACA mechanisms for reducing disparities can inform future policy responses targeting these remaining inequities.

## Data Availability

The datasets generated and/or analyzed during the current study are not publicly available due to articles being in subscription-based journals. However, they are available from the corresponding author on reasonable request.

## References

[1] Hill L, Artiga S, Haldar S. Key Facts on Health and Health Care by Race and Ethnicity. Kaiser Family Foundation; 2022 [cited 2022 Jul 25]. Available from: https://www.kff.org/racial-equity-and-health-policy/report/key-facts-on-health-and-health-care-by-race-and-ethnicity/

[2] National Center for Health Statistics. Health, United States, 2017: With Special Feature on Mortality. Hyattsville (MD): National Center for Health Statistics (US); 2018 [cited 2020 May 20]. (Health, United States). Available from: http://www.ncbi.nlm.nih.gov/books/NBK532685/30702833

[3] Suthers K. Evaluating the Economic Causes and Consequences of Racial and Ethnic Health Disparities. Issue Brief: American Public Health Association; 2008.

[4] Bound J, Waidmann T, Schoenbaum M, Bingenheimer JB (2003). The Labor Market Consequences of Race Differences in Health. Milbank Q..

[5] Michener J (2020). Race, Politics, and the Affordable Care Act. J Health Polit Policy Law..

[6] Braveman P, Gruskin S (2003). Defining equity in health. J Epidemiol Community Health..

[7] Snowden L, Graaf G (2019). The, “undeserving poor”, racial bias, and Medicaid coverage of African Americans. J Black Psychol..

[8] Orentlicher D (2015). Medicaid at 50: No Longer Limited to the “Deserving” Poor?. Yale J Health Policy Law Ethics..

[9] The Henry J. Kaiser Family Foundation. The Impact of the Coverage Gap in States not Expanding Medicaid by Race and Ethnicity. The Henry J. Kaiser Family Foundation; 2013.

[10] Artiga S, Damico A, Garfield RL. The Impact of the Coverage Gap for Adults in States not Expanding Medicaid by Race and Ethnicity. The Henry J. Kaiser Family Foundation; 2015 [cited 2018 Mar 19]. (The Kaiser Commission on Medicaid and the Uninsured). Available from: https://www.kff.org/disparities-policy/issue-brief/the-impact-of-the-coverage-gap-in-states-not-expanding-medicaid-by-race-and-ethnicity/

[11] Shamseer L, Moher D, Clarke M, Ghersi D, Liberati A, Petticrew M, et al. Preferred reporting items for systematic review and meta-analysis protocols (PRISMA-P) 2015: Elaboration and explanation. BMJ Br Med J Online 2015;349. [cited 2022 Mar 25] Available from: https://www.proquest.com/docview/1872328984/abstract/C13518D5DD82471EPQ/1 10.1136/bmj.g7647 25555855

[12] Guth M, Artiga S, Pham O. Effects of the ACA Medicaid Expansion on Racial Disparities in Health and Health Care. Kaiser Family Foundation; 2020 [cited 2021 Apr 21]. Available from: https://www.kff.org/report-section/effects-of-the-aca-medicaid-expansion-on-racial-disparities-in-health-and-health-care-appendix/

[13] Guth M, Ammula M. Building on the Evidence Base: Studies on the Effects of Medicaid Expansion, February 2020 to March 2021. Kaiser Family Foundation; 2021, 188.

[14] Menon A, Patel PK, Karmakar M, Tipirneni R (2021). The impact of the Affordable Care Act Medicaid expansion on racial/ethnic and sex disparities in HIV testing: National findings from the Behavioral Risk Factor Surveillance System. JGIM J Gen Intern Med..

[15] Johnson NL, Choi S, Herrera CN (2021). Black clients in expansion states who used opioids were more likely to access medication for opioid use disorder after ACA implementation. J Subst Abuse Treat..

[16] Ji X, Castellino SM, Mertens AC, Zhao J, Nogueira L, Jemal A, et al. Association of Medicaid expansion with cancer stage and disparities in newly diagnosed young adults. JNCI J Natl Cancer Inst. 2021;(djab105). [cited 2021 Jul 9] Available from: 10.1093/jnci/djab105 10.1093/jnci/djab105 PMC9989840 34021352

[17] Le Blanc JM, Heller DR, Friedrich A, Lannin DR, Park TS (2020). Association of Medicaid Expansion under the Affordable Care Act with breast cancer stage at diagnosis. JAMA Surg..

[18] Baumgartner JC, Collins SR, Radley DC, Hayes SL. How the Affordable Care Act has narrowed racial and ethnic disparities in access to health care. The Commonwealth Fund; 2020.

[19] Buchmueller TC, Levy HG (2020). The ACA’s impact on racial and ethnic disparities in health insurance coverage and access to care: An examination of how the insurance coverage expansions of the Affordable Care Act have affected disparities related to race and ethnicity. Health Aff (Millwood)..

[20] Artiga S, Orgera K, Damico A. Changes in Health Coverage by Race and Ethnicity since the ACA, 2010-2018. Kaiser Family Foundation. 2020; [cited 2020 Jul 6]. Available from: https://www.kff.org/disparities-policy/issue-brief/changes-in-health-coverage-by-race-and-ethnicity-since-the-aca-2010-2018/.

[21] Breslau J, Han B, Lai J, Yu H (2020). Impact of the Affordable Care Act Medicaid expansion on utilization of mental health care. Med Care..

[22] Wiggins A, Karaye IM, Horney JA (2020). Medicaid expansion and infant mortality, revisited: A difference-in-differences analysis. Health Serv Res..

[23] Barrington DA, Sinnott JA, Calo C, Cohn DE, Cosgrove CM, Felix AS (2020). Where you live matters: A National Cancer Database study of Medicaid expansion and endometrial cancer outcomes. Gynecol Oncol..

[24] Eliason EL (2020). Adoption of Medicaid Expansion Is Associated with Lower Maternal Mortality. Womens Health Issues..

[25] Brown CC, Moore JE, Felix HC, Stewart MK, Tilford JM (2020). County-level variation in low birthweight and preterm birth: An evaluation of state Medicaid expansion under the Affordable Care Act. Med Care..

[26] Glance LG, Thirukumaran CP, Shippey E, Lustik SJ, Dick AW (2020). Impact of Medicaid expansion on disparities in revascularization in patients hospitalized with acute myocardial infarction. PLoS ONE..

[27] Semprini J, Olopade O (2020). Evaluating the effect of Medicaid expansion on Black/White breast cancer mortality disparities: A difference-in-difference analysis. JCO Glob Oncol..

[28] Chaudry A, Jackson A, Glied SA. Did the Affordable Care Act reduce racial and ethnic disparities in health insurance coverage? The Commonwealth Fund; 2019 p. 11.

[29] Singh KA, Wilk AS (2019). Affordable Care Act Medicaid expansion and racial and ethnic disparities in access to primary care. J Health Care Poor Underserved..

[30] Lipton BJ, Decker SL, Sommers BD (2019). The Affordable Care Act appears to have narrowed racial and ethnic disparities in insurance coverage and access to care among young adults. Med Care Res Rev..

[31] Crocker AB, Zeymo A, McDermott J, Xiao D, Watson TJ, DeLeire T (2019). Expansion coverage and preferential utilization of cancer surgery among racial and ethnic minorities and low-income groups. Surgery..

[32] Wehby GL, Lyu W (2018). The impact of the ACA Medicaid expansions on health insurance coverage through 2015 and coverage disparities by age, race/ethnicity, and gender. Health Serv Res..

[33] Han X, Yabroff KR, Ward E, Brawley OW, Jemal A (2018). Comparison of insurance status and diagnosis stage among patients with newly diagnosed cancer before vs after implementation of the Patient Protection and Affordable Care Act. JAMA Oncol..

[34] Lee H, Porell FW (2018). The effect of the Affordable Care Act Medicaid expansion on disparities in access to care and health status. Med Care Res Rev..

[35] Yue D, Rasmussen PW, Ponce NA (2018). Racial/ethnic differential effects of Medicaid expansion on health care access. Health Serv Res..

[36] Hayes S, Riley P, Radley D, McCarthy D. Reducing racial and ethnic disparities in access to care: Has the Affordable Care Act made a difference?. New York: Commonwealth Fund; 2017 [cited 2020 May 20]. Available from: http://www.issuelab.org/permalink/download/28158 28836751

[37] Flores RD, Vargas R (2017). Medicaid expansion and ethnoracial disparities in health insurance coverage. J Ethn Migr Stud..

[38] Buchmueller TC, Levinson ZM, Levy HG, Wolfe BL (2016). Effect of the Affordable Care Act on racial and ethnic disparities in health insurance coverage. Am J Public Health..

[39] McMorrow S, Long SK, Kenney GM, Anderson N (2015). Uninsurance disparities have narrowed for Black and Hispanic adults under the Affordable Care Act. Health Aff (Millwood)..

[40] Agarwal A, Katz AJ, Chen RC (2019). The Impact of the Affordable Care Act on disparities in private and Medicaid insurance coverage among patients under 65 with newly diagnosed cancer. Int J Radiat Oncol..

[41] Han X, Jemal A, Zheng Z, Sauer AG, Fedewa S, Yabroff KR. Changes in noninsurance and care unaffordability among cancer survivors following the Affordable Care Act. JNCI J Natl Cancer Inst. 2019;djz218. 10.1093/jnci/djz218 PMC7357320 31688923

[42] Xiao D, Zheng C, Jindal M, Johnson LB, DeLeire T, Shara N (2018). Medicaid Expansion and Disparity Reduction in Surgical Cancer Care at High Quality Hospitals. J Am Coll Surg..

[43] Messer LC. Invited commentary: measuring social disparities in health--what was the question again? Am J Epidemiol. 2008;167(8):900–904; author reply 908-916. 10.1093/aje/kwn019 18344512

[44] Moonesinghe R, Beckles GLA (2015). Measuring health disparities: A comparison of absolute and relative disparities. PeerJ..

[45] Harper S, Lynch J. Methods for Measuring Cancer Disparities: Using Data Relevant to Healthy People 2010 Cancer-Related Objectives. 2005 [cited 2022 Jul 26]; Available from: https://drum.lib.umd.edu/handle/1903/22744

[46] Brooks T, Miskell S, Artiga S, Cornachione E, Gates A. Medicaid and CHIP Eligibility, Enrollment, Renewal, and Cost-Sharing Policies as of January 2016: Findings from a 50-State Survey. The Henry J. Kaiser Family Foundation; 2016 p. 74. (The Kaiser Commission on Medicaid and the Uninsured).

[47] Graetz I, Kaplan CM, Kaplan EK, Bailey JE, Waters TM (2014). The U.S. health insurance marketplace: Are premiums truly affordable?. Ann Intern Med..

[48] Goldman AL, McCormick D, Haas JS, Sommers BD (2018). Effects of the ACA’s health insurance marketplaces on the previously uninsured: A quasi-experimental analysis. Health Aff (Millwood)..

[49] Goldman AL, Woolhandler S, Himmelstein DU, Bor DH, McCormick D (2018). Out-of-pocket spending and premium contributions after implementation of the Affordable Care Act. JAMA Intern Med..

[50] Andrulis D, Siddiqui N, Purtle J, Duchon L (2010). Patient Protection and Affordable Care Act of 2010: Advancing Health Equity for Racially and Ethnically Diverse Populations.

[51] Rosenbaum S (2017). The Community Health Center Fund: What’s at risk?. Milbank Q..

[52] Sonier J, Boudreaux MH, Blewett LA (2013). Medicaid ‘Welcome-Mat’ effect of Affordable Care Act implementation could be substantial. Health Aff (Millwood)..

[53] Denham A, Veazie P (2019). Did Medicaid expansion matter in states with generous Medicaid?. Am J Manag Care..

[54] Graaf G, Snowden L (2020). Medicaid waiver adoption for youth with complex behavioral health care needs: An analysis of state decision-making. J Disabil Policy Stud..

[55] Wright B, Askelson NM, Ahrens M, Momany E, Bentler S, Damiano P (2017). Completion of requirements in Iowa’s Medicaid expansion premium disincentive program, 2014–2015. Am J Public Health..

[56] Zylla E, Planap C, Lukanen E, Blewett LA. Section 1115 Medicaid Expansion Waivers: Implementation Experiences: MACPAC. State Health Access Data Assistance Center; 2018 [cited 2019 Dec 9]. Available from: https://www.macpac.gov/publication/section-1115-medicaid-expansion-waivers-implementation-experiences/

[57] Sommers BD, Tomasi MR, Swartz K, Epstein AM (2012). Reasons for the wide variation in Medicaid participation rates among states hold lessons for coverage expansion in 2014. Health Aff Proj Hope..

[58] Sommers BD, Blendon RJ, Orav EJ (2016). Both the ‘private option’ and traditional Medicaid expansions improved access to care for low-income adults. Health Aff (Millwood)..

[59] Musumeci M. Disability and Technical Issues Were Key Barriers to Meeting Arkansas’ Medicaid Work and Reporting Requirements in 2018. Henry J. Kaiser Family Foundation. 2019:12.

[60] Watkins-Hayes C. Race, respect, and red tape: Inside the black box of racially representative bureaucracies. J Public Adm Res Theory. 2011;21(suppl_2):i233–51.

[61] Kaiser Family Foundation P. Medicaid Waiver Tracker: Approved and Pending Section 1115 Waivers by State. 2019. [cited 2019 Dec 10]. Available from: https://www.kff.org/medicaid/issue-brief/medicaid-waiver-tracker-approved-and-pending-section-1115-waivers-by-state/

[62] Einav L, Finkelstein A (2018). Moral hazard in health insurance: What we know and how we know it. J Eur Econ Assoc..

[63] Williams DR, Priest N, Anderson NB (2016). Understanding associations among race, socioeconomic status, and health: Patterns and prospects. Health Psychol..

[64] Blumenthal D, Morone J (2010). The Heart of Power.

[65] Lerman AE, McCabe KT (2017). Personal experience and public opinion: A theory and test of conditional policy feedback. J Polit..

[66] Oberlander JB, Lyons B (2009). Beyond incrementalism? SCHIP and the politics f health reform. Health Aff (Millwood)..

